# Federated Learning-Based Insulator Fault Detection for Data Privacy Preserving

**DOI:** 10.3390/s23125624

**Published:** 2023-06-15

**Authors:** Zhirong Luan, Yujun Lai, Zhicong Xu, Yu Gao, Qian Wang

**Affiliations:** School of Electrical Engineering, Xi’an University of Technology, Xi’an 710048, China; 2221920082@stu.xaut.edu.cn (Y.L.); xuzhicong@stu.xaut.edu.cn (Z.X.); 3200211055@stu.xaut.edu.cn (Y.G.); wangqian77@xaut.edu.cn (Q.W.)

**Keywords:** vision sensor, insulator fault detection, federated learning, privacy-preserving

## Abstract

Insulators are widely used in distribution network transmission lines and serve as critical components of the distribution network. The detection of insulator faults is essential to ensure the safe and stable operation of the distribution network. Traditional insulator detection methods often rely on manual identification, which is time-consuming, labor-intensive, and inaccurate. The use of vision sensors for object detection is an efficient and accurate detection method that requires minimal human intervention. Currently, there is a considerable amount of research on the application of vision sensors for insulator fault recognition in object detection. However, centralized object detection requires uploading data collected from various substations through vision sensors to a computing center, which may raise data privacy concerns and increase uncertainty and operational risks in the distribution network. Therefore, this paper proposes a privacy-preserving insulator detection method based on federated learning. An insulator fault detection dataset is constructed, and Convolutional Neural Network (CNN) and Multi-Layer Perceptron (MLP) models are trained within the federated learning framework for insulator fault detection. Most of the existing insulator anomaly detection methods use a centralized model training method, which has the advantage of achieving a target detection accuracy of over 90%, but the disadvantage is that the training process is prone to privacy leakage and lacks privacy protection capability. Compared with the existing insulator target detection methods, the proposed method can also achieve an insulator anomaly detection accuracy of more than 90% and provide effective privacy protection. Through experiments, we demonstrate the applicability of the federated learning framework for insulator fault detection and its ability to protect data privacy while ensuring test accuracy.

## 1. Introduction

In distribution networks, insulators are very important components and play a major role in the safe and stable operation of the distribution network [1]. However, due to their long-term exposure to outdoor environments, insulators are susceptible to faults and damage. Failure to timely detect such issues can lead to a shortened lifespan of insulators, adversely impacting the transmission efficiency of power systems and even giving rise to hazardous events such as fires and explosions [2]. Therefore, it is of great significance to ensure that the insulators are in good working condition in the distribution network, regularly detect insulator faults, find insulator defects, and eliminate faults in time for the safe and stable operation of the distribution network. Traditional fault detection methods primarily involve manual observations of insulators by maintenance personnel to identify external defects. This labor-intensive and subjective approach not only incurs substantial labor costs but also results in subjective misjudgments, compromising the effectiveness of the detection process [3].

Computer vision is a field that utilizes computer algorithms to process and interpret digital images or videos [4]. It involves various disciplines, such as image processing, pattern recognition, computer graphics, and machine learning, with the aim of enabling computers to understand and analyze images or videos in a similar manner to humans by using vision sensors. Object detection, an important task in computer vision [5], has been widely applied in industrial inspection in recent years. By employing object detection algorithms, it becomes possible to identify surface defects such as cracks, deformations, and component losses in industrial products, thereby improving product quality and production efficiency. Vision sensors play a crucial role in object detection, as they can automatically capture images and video data from the environment using visual capture devices such as cameras. The captured image data are pre-processed to reduce image noise, thereby improving the accuracy and efficiency of object detection [6].

In this paper, vision sensors are used to capture images of insulators. Object detection techniques are applied to automate and achieve high-precision detection of insulators [7], enabling the detection of defects and damages that are difficult to perceive with the naked eye. Compared to manual inspection, this approach is more efficient, reliable, and avoids hazardous tasks such as working at height and handling high voltages. In addition, vision sensors can capture many insulator images within a short period. By applying object detection algorithms for multiple inspections and recognition of insulator faults, the approach can mitigate the impact of occasional factors that may lead to misjudgment, ensuring the reliability of the detection results. Therefore, the use of object detection methods based on vision sensors for insulator fault detection has significant value and broad application potential [8].

Nowadays, insulators are widely used in distribution network transmission lines, and the vision sensor systems of substations in different distribution networks can only capture images within their respective areas, resulting in limited data availability [9]. To utilize intelligent image detection methods for identifying insulator faults, it is necessary to upload data from various locations to a centralized computing center for model training. However, this centralized data uploading approach carries the risk of privacy issues and potential data breaches. Considering this, it is imperative to research a privacy-preserving method for insulator fault detection.

The concept of federated learning was first introduced in the literature [10] in 2017, proposing the use of the Federated Averaging (FedAvg) algorithm to achieve distributed dataset training and providing a comprehensive description of its application scenarios. Federated learning is a machine learning method that leverages the idea of distributed computing, enabling multiple edge devices to perform model training locally and only upload model parameters to a central server. The central server aggregates all model parameters, updates the global model, and distributes it back to the participating devices, facilitating collaborative learning. This approach keeps the data localized without the need for data uploading to the central server, thereby enhancing data privacy and security [11].

**Figure 1:**
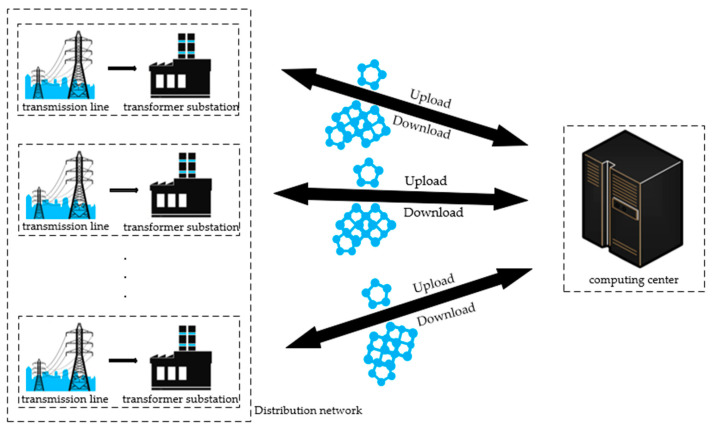
Schematic diagram of distributed insulator fault detection based on federated learning. Each substation collects insulator image information from local power transmission lines. Subsequently, a dataset is constructed, and a local model is trained using this dataset. The local model is then uploaded to the computing center. The computing center aggregates and updates all uploaded local models and distributes the generated global model to each substation.

Based on the above analysis, aiming at the insulator fault problem in the distribution network, this paper proposes a distributed insulator fault detection method based on federated learning. Considering the privacy-preserving capabilities of federated learning, substations in different distribution networks conduct local training on their insulator datasets to obtain local models, which are then uploaded to the central computing center. The computing center aggregates and updates all local models, generating a global model that is subsequently distributed to the participating substations. This process is shown in Figure 1. This distributed federated learning approach not only achieves model sharing but also mitigates privacy concerns associated with uploading data to a central computing center. Moreover, it maintains an acceptable level of model performance.

The originality of this work lies in the fact that this paper proposes a distributed insulator fault detection method based on federal learning. No other similar work has been carried out to maintain high accuracy of detection under privacy-preserving conditions, and the core of this paper is to protect data privacy during the target detection. Existing insulator fault detection methods collect raw data in a cloud server to train the model, a process that inevitably compromises privacy. The method used in this paper places the model training process on the local server and uploads the trained model from the local server to the cloud server. This process only uploads the model data to the cloud server without uploading the raw data, which avoids uploading the raw data directly to the cloud server and solves the privacy problem well. Good insulator detection accuracy is maintained.

This paper presents the construction of an insulator fault detection dataset and explores the impact of different data distributions on the performance of insulator fault detection. The dataset comprises over 4000 images, with 5 label types [12]. To investigate the influence of data distribution, two types of datasets were created: the overall dataset and the independently identically distributed (IID) dataset. The overall dataset includes all image data and simulates the process of training a centralized model in the computing center, serving as the baseline for this study. The IID dataset was created by horizontally partitioning the data of each training node based on samples, ensuring that each node contains complete features and data labels, following an independent and identically distributed pattern.

In Section 5.1, we constructed Multilayer Perceptron (MLP) and Convolutional Neural Network (CNN) models based on the federated learning framework. The Federated Averaging (FedAvg) aggregation method was used to aggregate the parameters of each agent at the computing center, and the aggregated model was distributed back to each agent for local model updates. A distributed cost function based on cross-entropy was constructed, and stochastic gradient descent was employed to search for the optimal solution of the model [13].

Furthermore, we validated the accuracy of the federated learning approach through experiments. Two types of neural networks, MLP and CNN, were utilized to train models for insulator fault detection in both the traditional experiment and the federated learning experiment [14]. The accuracy of federated learning was compared and verified. The experimental results demonstrated that federated learning achieved acceptable accuracy compared to the centralized mode, while ensuring data privacy protection without compromising model precision.

In conclusion, the main contributions of this work are as follows: (1) the construction of a dataset for distributed insulator fault detection, specifically designed for training federated learning models, (2) the implementation of a federated learning approach for distributed insulator fault detection model training and testing, and (3) the transmission of only model parameters between agents and the computing center, avoiding the privacy risks associated with data uploading.

The remainder of the paper is organized as follows: Section 2 presents related work, Section 3 describes preliminary work on insulator target detection algorithms, Section 4 constructs an insulator detection dataset, Section 5 provides a federal learning-based insulator fault detection algorithm, Section 6 presents the experimental results, and Section 7 and Section 8 discuss and summarize the methods proposed in this paper and the contributions, respectively.

## 2. Related Work

### 2.1. Insulator Detection

With the advancement and development of object detection technology, this method has been widely applied in the field of industrial inspection, playing an important role in the recognition and detection of devices such as insulators. Numerous studies have been conducted on object detection-based insulator fault detection methods.

Wang et al., 2016 propose a novel method for insulator recognition in aerial images captured by drones in highly cluttered backgrounds [15]. This method utilizes the machine learning algorithm Support Vector Machine (SVM) as a classifier and employs Gabor features to distinguish insulators from cluttered backgrounds. This approach not only achieves successful insulator recognition in complex and cluttered aerial images but also significantly reduces the computational complexity and minimizes misclassification through background suppression. Xing et al., 2022 propose a lightweight model based on MobileNet-YOLOv4, which incorporates Gaussian filtering, K-Means++ clustering, and Mosaic data augmentation for dataset preprocessing to improve the detection accuracy [16]. The proposed model is tested for insulator recognition in power transmission lines, and the results show that it outperforms YOLOv4 in terms of recognition accuracy, average detection speed, and model size, meeting the requirements of real-time monitoring. An improved Fast Region-based Convolutional Neural Network (R-CNN)-based method for insulator detection in image detection has also been published [17]. The anchor generation method and non-maximum suppression (NMS) in the Region Proposal Network (RPN) of the Faster R-CNN model are studied and improved. Experimental results demonstrate a significant enhancement in insulator detection, and the improved NMS effectively distinguishes and detects occluded insulators. An effective and reliable insulator detection method based on aerial image deep learning technology is proposed in [18]. This method utilizes the Single-Shot Multi-box Detector (SSD) model for automatic multi-level feature extraction in aerial images and combines fine-tuning the COCO model with aerial images to obtain a basic insulator model. Furthermore, the basic model is fine-tuned using a training set consisting of specific insulator types and specific detection scenarios. The results show a significant improvement in accuracy, efficiency, and robustness. A deep learning-based approach is proposed for detecting insulator fractures in complex conditions in [19]. The Region-based Fully Convolutional Network (R-FCN) is employed. Firstly, image rectification and cropping techniques are applied to images with different sample sizes and orientations. Secondly, image augmentation techniques are utilized to expand the dataset. Finally, a sliding window and coordinate mapping method are employed during the testing phase. The established model exhibits strong robustness and environmental adaptability.

All five of the above insulator detection methods use a centralized detection approach that requires the collection of data from all clients, and all these works have the disadvantage of not having privacy protection capabilities. In contrast, federation learning allows model training without sharing client data, effectively protecting user privacy.

### 2.2. Federated Learning

Considering the data privacy protection features of distributed methods, extensive research has been conducted both domestically and internationally in this area. The authors of [10] proposed a new method called federated learning, to address the issue of model training on distributed datasets on mobile devices. Each device, i.e., client, trains its local dataset and only exchanges model updates with the server, thereby significantly protecting data privacy. As federated learning aggregates model updates instead of raw data, it plays a prominent role in privacy security. However, there is still a possibility of raw data leakage. Geyer et al., 2017 proposed to implement differential privacy on the central server to enhance privacy protection [20]. Zhu et al., 2021 introduced the effects of a heterogeneous system, heterogeneous data distribution, and a heterogeneous model under different environments and tasks on federated learning performance. Additionally, they proposed a knowledge distillation method that can address the challenges of heterogeneous federated learning without sharing data [21]. The aforementioned research on federated learning indicates that this distributed method can effectively address the issue of data leakage when uploading data to a central server and protect client data privacy. Additionally, the federated learning method eliminates the need to transfer data to the central server, reducing the amount of data transmission. Moreover, it allows online learning, enabling real-time model sharing and continuous updates. Building upon the foundation of the aforementioned work, we considered proposing a federated learning-based solution for insulator object detection.

All the above work has good privacy protection capabilities, and the federal learning approach can effectively address the risk of privacy breaches. The methods used in the above work can address the privacy protection issues of insulator fault detection.

## 3. Preliminary Work

Federated learning is a distributed machine learning method, which can perform distributed learning on local datasets under the premise of protecting data privacy, to realize the creation of global machine learning models [22].

### 3.1. Federated Learning Framework

The structure of federated learning mainly includes four parts: local nodes, computing centers, models, and federated learning algorithms. Local nodes are individuals in a distributed system, and each node contains databases and computing resources for storing and processing data. In this paper, the local node refers to the local substation for insulator data training, and the computing center is the central server responsible for the management of the training process and the update of the machine learning model. In this paper, the computing center aggregates the models uploaded by the local nodes, updates them into a global model, and then distributes them to each local node, and each local node runs a machine learning model for local data training and local model updating. The target detection algorithm based on deep learning is used to train the local insulator fault detection model, which can detect the insulator faults collected by the local substation. The federated learning algorithm is a special machine learning algorithm that can aggregate local models and update them into global models for distribution [23]. It can be used by any local node while avoiding the privacy leakage problem caused by sharing the original data.

### 3.2. Centralized Model

**Figure 2:**
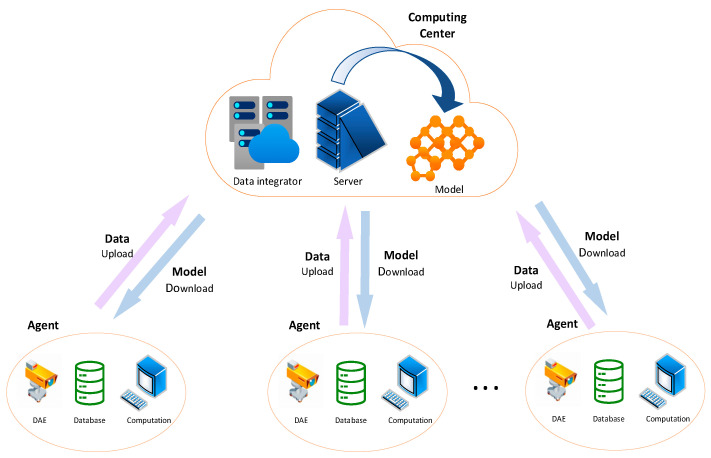
Centralized model. Each client collects local image data, uploads all the image data to the computing center, and the computing center uniformly trains the global model and distributes the global model to each local client.

In this paper, the centralized model obtained by the training of the overall dataset was used as the baseline of this research [24]; that is, the global model was obtained by using the overall dataset for training without splitting the local dataset. This scheme simulates the process of uploading all the insulator image data of each substation to the computing center and performing unified training in the computing center to obtain a global model trained by all insulator datasets, and then sending this model back to each substation for fault detection. This process is illustrated in Figure 2.

### 3.3. Federated Learning Model

**Figure 3:**
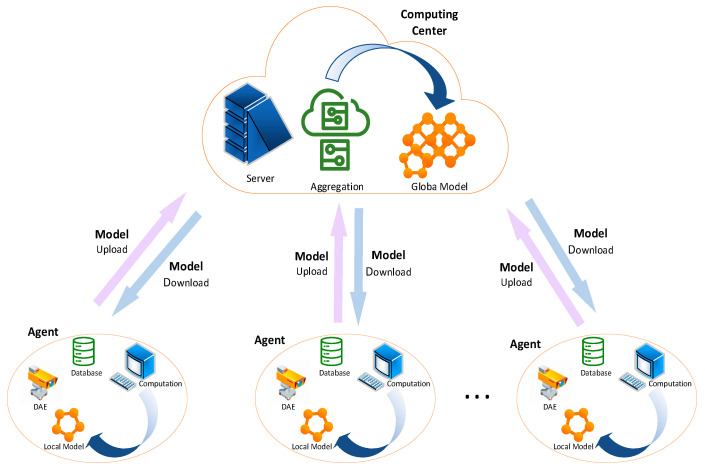
Federated learning model. Each client collects image data information locally, builds a dataset to train a local model, the client uploads the local model to the computing center, and the computing center integrates and updates to generate a global model and distributes it to each client.

The federated learning model was obtained by using a distributed machine learning method to obtain a local model from a local training dataset, and then uploading the local model to the computing center for aggregation and updating. The federated experiment simulates that each substation trains its own insulator dataset locally [25], then uploads the trained local model to the computing center, aggregates all the local models in the computing center, updates them into a global model, and then distributes the global model to each grassroots substation process. This process is illustrated in Figure 3. In this way, model sharing, and multi-party collaboration training, can be realized under the premise of privacy protection.

### 3.4. Federated Learning Optimization Algorithm

However, a deployed federated optimization system also has to deal with many practical issues, such as the changing insulator datasets as data is added and removed. In the optimization simulation experiment in this paper, the computing center randomly selects a subset of substations, sends the current global algorithm state (such as the current model parameters) to these substations, and then each selected local substation will compute locally according to the global state and the local insulator dataset, and send result updates back to the computing center. The computing center applies these updates to the global state and proceeds to the next round. This approach enables distributed optimization while avoiding the disadvantages of data privacy leaks and high communication costs in centralized training.

The algorithm considered in this paper is applicable to any objective function of the form finite sum.

We define a loss function $f\left(\omega \right)$. $f\left(\omega \right)$ is expressed as the average of the loss function $f_{i}\left(\omega \right)$ for all n data samples. The loss function $f_{i}\left(\omega \right)$ of each data sample represents the difference or error between the predicted result of the i-th data sample and the true label under the given model parameters, ω. By summing the loss functions for all data samples and dividing by the total number of data samples n, the average loss function, $f\left(\omega \right)$, is obtained. We used this formula to calculate the average loss of the model over all data samples, thereby measuring the performance of the model on the entire dataset [10], as shown in Formula (1):

(1) $$\underset{\omega \in R^{d}}{min}f\left(\omega \right)\;\;s.t.\;\;f\left(\omega \right)≝\frac{1}{n}\sum_{i=1}^{n}f_{i}\left(\omega \right)$$

### 3.5. Federated Learning Privacy Shield

Federated learning is a distributed machine learning approach that allows participants to train models on their local devices without directly sharing raw data. It achieves collaborative learning among participants through model sharing and aggregation [26]. Federated learning offers capabilities in privacy protection, such as data decentralization, local data processing, encrypted communication, model aggregation, and differential privacy. Data decentralization refers to the fact that federated learning does not require uploading the raw dataset to a central server. Sensitive data on the client devices can be protected locally, avoiding privacy risks associated with centralizing the dataset. Local data processing means that data processing and model training are performed on the local devices in federated learning. Clients can control and protect their data locally and decide when to transmit the model to the central server. Encrypted communication means that federated learning utilizes encryption protocols for participants to exchange model updates, ensuring the confidentiality of data during the transmission of model updates [27]. Model aggregation refers to the process in federated learning where local models uploaded by clients to the central server are merged using aggregation algorithms to generate a global model. The aggregation process is performed in an encrypted state, ensuring that each client cannot access other models, apart from their local models, thus protecting data privacy among clients. Additionally, federated learning can employ differential privacy to protect client data. By introducing noise during the model training process, the individual contributions of personal data are hidden, reducing the risk of sensitive information leakage [28].

## 4. Construction of Insulator Fault Detection Dataset

To apply a distributed federated learning method to detect insulator faults, we constructed an insulator fault detection dataset [29]. First, we collected more than 1000 insulator images and screened them in terms of resolution and similarity to ensure the image quality of the dataset. Second, since the insulator dataset contains rich data information, we labeled the images in the dataset into five categories, and each image had a complete attribute and feature label. Third, we split the insulator dataset. We divided the dataset into 10 independent and identically distributed small datasets and used the average value of the model training parameters of each small dataset as the final dataset index.

### 4.1. Image Collection

The insulator dataset mainly comes from the detection equipment of substations at various locations. In addition to the images taken by the detection equipment, we also collected a variety of insulator fault images from the website for dataset expansion and content supplementation.

In this paper, there were more than 4000 images of insulators taken by testing equipment in substations in various places, covering different regions and types of insulators. The degree of contamination or damage to insulators in different regions is closely related to the environmental conditions of each region. The differences in air humidity, atmospheric wind speed, rainfall intensity, and other indicators will cause different degrees of damage to insulators [30]. Therefore, we selected insulator images taken by substations in different regions for dataset construction, which is more representative, can avoid the influence of accidental factors, and can improve the accuracy of the target detection model.

Since most of the images taken by substations in various places were taken in real time by testing equipment, the transmission lines were in normal operation, and the insulators on the towers were mostly in normal conditions, or only a few places had slight wear and tear, and the damage was relatively small. Therefore, in order to expand the type of dataset and obtain images of insulators with greater damage, we used the image library to search for keywords and collected more than 1000 insulator fault images on the website. In the end, more than 5000 images of different types of insulators were collected.

In this paper, the collected images were compared for similarity using mean square error (MSE), counting the number of pixels in an image, and identifying images with high similarity or duplication [31]. In addition, we counted the resolution of images, and deleted images with low resolution, poor image quality, and repeated or high similarity. Finally, more than 4000 images were retained as the total dataset.

### 4.2. Image Annotation

**Figure 4:**
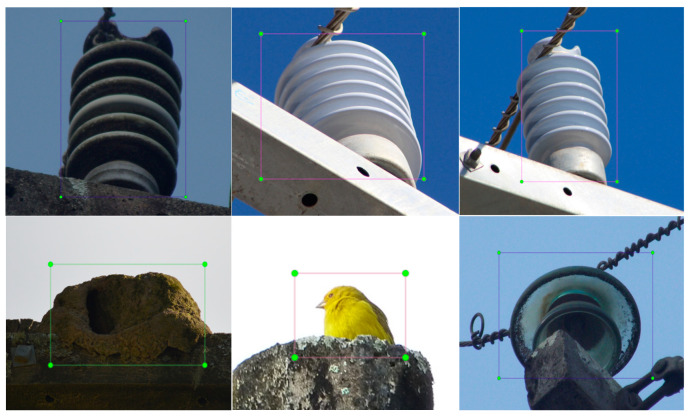
Target annotation image.

We used the LabelImg to complete the target labeling of each picture collected [32]. A total of five label categories of “defective insulator”, “nest”, “grass”, “bird”, and “normal insulator” were marked. The target annotation image is shown in Figure 4.

The result of labeling with LabelImg is to generate a label file corresponding to the picture one-by-one and generate a label directory file. These two types of files were saved together in the previously created annotation file. The label directory file indicated that 0, 1, 2, 3, and 4 correspond to five different label types, of “defective insulator”, “nest”, “grass”, “bird”, and “normal insulator”, respectively. In the label file generated for each picture, the first number indicates the label category, 0 for “defective insulator”, 1 for “nest”, 2 for “grass”, 3 for “bird”, and 4 for “normal insulator”, while the last four numbers represent the coordinate position, the first two coordinates represent the center point, and the last two coordinates represent the length and width.

### 4.3. Dataset Segmentation

In order to evaluate the performance of the distributed federated learning method for identifying insulator faults, we used two experimental methods, the baseline experiment and the federated experiment, for comparative analysis. The overall dataset and the independent and identically distributed dataset were required, so we divided the dataset to obtain independent and identically distributed datasets.

In this paper, the K-fold cross-validation method was used to split the dataset. K-fold cross-validation is a commonly used method for evaluating machine learning models [33]. It divides the dataset into k subsets of equal size, and then uses each subset in turn as test data, and the remaining k-1 subsets as training data, repeating this process k times, each time using a different subset as test data, and finally, obtaining the average of the performance evaluation results of k models as the final performance evaluation of the model.

K-fold cross-validation reduces the risk of overfitting. Each evaluation uses a different subset of the dataset for training and verification. Multiple evaluations are performed on the training data, which reduces the error caused by the randomness of the data. It can accurately evaluate the performance of the model on unseen data and effectively improve the generalization ability of the model. K-fold cross-validation compares the performance of different models on the same dataset and selects the best model. In addition, K-fold cross-validation utilizes all data for training and validation to maximize the use of the data [34].

The specific steps of using K-fold cross-validation in this paper are as follows:

(1) Randomly shuffle the order of images in the insulator dataset to ensure that the images in each dataset after segmentation are random.
(2) Divide the images in the insulator dataset into 10 parts, and each part contains as many images as possible.
(3) For each piece of data, use it as a verification set, and the remaining nine pieces of data as a training set, then train a model and record its performance indicators.
(4) Repeat step 3 continuously to ensure that each piece of data is used as a verification set, and a total of 10 training and testing sequences are performed.
(5) Take the average of the model performance indicators obtained from each training as the final performance indicator.

## 5. Proposed Federated Learning-Based Insulator Fault Detection

### 5.1. Model Construction

#### 5.1.1. MLP Model Construction

Here, we present the construction of an MLP model for insulation fault detection. MLP, which stands for Multilayer Perceptron, is a type of feedforward neural network. It consists of multiple neurons organized into several layers. In the constructed MLP model, each neuron receives multiple inputs, performs a linear combination of these inputs, and adds a bias term.

(2) $$z_{j}^{\left(l\right)}=\sum_{i=1}^{n^{\left(l-1\right)}}w_{ij}^{\left(l\right)}a_{i}^{\left(l-1\right)}+b_{j}^{\left(l\right)}$$

In Formula (2), l−1 represents the number of neurons in layer i−1, $w_{ij}^{\left(l\right)}$ represents the connection weight between the *i*-th neuron of layer l−1 and the j-th neuron of layer l, $a_{i}^{\left(l-1\right)}$ represents the input of the i-th neuron of the l−1-th layer, $b_{j}^{\left(l\right)}$ represents the bias item of the j-th neuron of the l-th layer, and $z_{j}^{\left(l\right)}$ represents the linear combination input received by the j-th neuron of the l-th layer. In this formula, the sum of the product of each input, $a_{i}^{\left(l-1\right)}$, and the connection weight, $w_{ij}^{\left(l\right)}$, plus the calculation process of the bias element, $b_{j}^{\left(l\right)}$, is the linear transformation of the neuron, which maps the input data to a new space [35].

The ReLU function is a commonly used activation function, which maps the input value to the output value. The mathematical expression of the ReLU function is as follows:

(3) f(x)=max⁡(0,x)

or segmented as:

(4) $$f(x)=\left\{\begin{array}{cc} 0, & x\leq 0\\ x, & x>0 \end{array}\right.$$

The role of the ReLU function is to introduce a non-linear mapping to the output of the neuron, thereby increasing the expressiveness of the network [36]. When the input x is greater than 0, the derivative of the ReLU function is 1. When the input x is less than or equal to 0, the derivative of the ReLU function is 0. This property makes the training of the network more stable, avoiding the problems of gradient vanishing and exploding.

Therefore, the output of each neuron can be expressed as:

(5) $$h(x)=f(\sum_{i=1}^{n}w_{i}x_{i}+b)$$

In this context, $x_{i}$ represents the i input, $w_{i}$ represents the weight corresponding to the i input, b represents the bias, and f represents the activation function. In an MLP, each neuron is typically assigned to different layers, and there are connections between each layer. The input to the next layer is the output of the previous layer. Typically, an MLP consists of at least one input layer, one output layer, and one or more hidden layers.

In this paper, cross-entropy was constructed as the cost function of MLP, and cross-entropy was used to measure the difference between the model output and the real label [37]. For a classification problem with C categories, the mathematical expression for cross-entropy is:

(6) $$\mathrm{CE}(y,\hat{y})=-\sum_{i=1}^{C}y_{i}\log({\hat{y}}_{i})$$

where $y=(y_{1},y_{2},\cdots ,y_{C})$ represents the true label, and ${\hat{y}}_{i}=({\hat{y}}_{1},{\hat{y}}_{2},\cdots ,{\hat{y}}_{c})$ indicates the predicted value of the model. In Formula (6), when the i element of the real label y is 1, the corresponding cross-entropy calculation term is $\log({\hat{y}}_{i})$; otherwise, it is 0. The result of the cross-entropy calculation is a scalar that represents the difference between the model’s predicted value and the true label.

The predicted value of the model was output by the SoftMax function in this paper. The mathematical expression of the SoftMax function is:

(7) $${\hat{y}}_{i}=\frac{\exp(z_{i})}{\sum_{j=1}^{C}exp(z_{i})}$$

where ${\hat{y}}_{i}$ represents the probability that the model predicts the i-th category. Through the output of the SoftMax function, the output of the model can be converted into a probability distribution, such that each output value is in the range [0, 1] and the sum of all output values is 1.

The core algorithm of MLP is the backpropagation algorithm, which is used to optimize the weights and biases of the model so that the performance of the model on the training data is as close as possible to the actual situation. The basic idea of the backpropagation algorithm is to update the weights and biases by calculating the partial derivatives of the cost function with respect to the weights and biases.

The backpropagation algorithm is divided into two stages: forward propagation and backpropagation. For the forward propagation stage, the input sample is fed into the network, calculated layer-by-layer according to the hierarchical structure, and the output result is obtained. For the backpropagation stage, according to the partial derivative of the cost function on the weight and bias, the error is calculated backwards layer-by-layer, and the weight and bias of each neuron are updated [38]. The parameters in the network are updated using the gradient descent method to minimize the cost function (loss function).

The specific steps of the backpropagation algorithm are as follows:

In the forward propagation stage, the weighted input, $z^{\left[l\right]}$, and activation value, $a^{\left[l\right]}$, of each layer are calculated, and the final prediction value, $\hat{y}$, is output.

(8) $$z^{\left[l\right]}=w^{\left[l\right]}a^{\left[l-1\right]}+b^{\left[l\right]}$$

(9) $$a^{\left[l\right]}=\sigma (z^{\left[l\right]})$$

where, $a^{\left[l\right]}$ represents the activation value of the input layer, that is, the input feature of the model.

Compute the error, $\delta^{\left[L\right]}$, for the output layer:

(10) $$\delta^{\left[L\right]}=\hat{y}-y$$

Backpropagation error:

(11) $$\delta^{\left[l\right]}=(w^{\left[l+1\right]}{)}^{T}\delta^{\left[l+1\right]}⊙\sigma^{\prime }(z^{\left[l\right]})$$

where ⊙ means element-wise multiplication, and $\sigma^{\prime }(z^{\left[l\right]})$ means the derivative of the layer l activation function.

Compute the gradient of the cost function with respect to the weight matrix and bias vector of layer l:

(12) $$\frac{\partial J}{\partial w^{\left[l\right]}}=\delta^{\left[l\right]}(a^{\left[l-1\right]}{)}^{T}$$

(13) $$\frac{\partial J}{\partial b^{\left[l\right]}}=\delta^{\left[l\right]}$$

Update the parameters in the network using gradient descent:

(14) $$w^{\left[l\right]}:=w^{\left[l\right]}-\alpha \frac{\partial J}{\partial w^{\left[l\right]}}$$

(15) $$b^{\left[l\right]}:=b^{\left[l\right]}-\alpha \frac{\partial J}{\partial b^{\left[l\right]}}$$

where α represents the learning rate, which controls the step size of the parameter update. By continuously iteratively performing the above steps, the cost function can be minimized and the parameters in the neural network can be updated, thereby improving the performance of the model.

#### 5.1.2. CNN Model Construction

We constructed a mathematical model of CNN, which can be expressed as a multi-layer neural network, where each layer consists of multiple convolution kernels and a nonlinear activation function. Each convolution kernel is a set of weights that slide over the input image and perform a convolution operation to extract features. Each convolution kernel performs the same operation at each position of the input image and produces a feature map. These feature maps are passed to the next layer for the next round of convolution operations [39].

Specifically, set the input image to be a two-dimensional matrix X with size w∗h, where w is the width of the image and h is the height of the image. Suppose there are k convolution kernels in the l-th layer, and the size of each convolution kernel is $w_{l}*h_{l}$, where $w_{l}$ is the width of the convolution kernel and $h_{l}$ is the height of the convolution kernel. Then, the output feature map of the first layer can be expressed as a three-dimensional matrix $Y_{l}$, $Y_{l}$ for each element $Y_{l,i,j,k}$, and its value can be expressed as:

(16) $$Y_{l,i,j,k}=f(\sum_{p=1}^{w_{l}}\sum_{q=1}^{h_{l}}X_{i+p-1,j+q-1}{\;W}_{i,p,q,k}+b_{k})$$

where $W_{l,i,j,k}$ is the weight of row p and column q of the k-th convolution kernel of the layer l, $b_{k}$ is the bias item of the k-th convolution kernel, and f is a nonlinear activation function, such as ReLU, etc. In addition, CNN usually adds a pooling layer after several layers to reduce the size of the feature map and extract higher-level image features.

To reduce dimensionality and extract higher-level features, we added additional pooling layers. The purpose of the pooling layer is to reduce the size of the feature map and enhance the translation invariance of the features. It can reduce the size of the feature map by taking the maximum value of each local region in the feature map as its output to reduce the number of parameters and the computation of the model.

The mathematical expression of maximum pooling is:

(17) $$Y_{i,j}={max}_{m=1}^{p}{max}_{n=1}^{p}X_{(i-1)\times s+m,(j-1)\times s+n}$$

where $Y_{i,j}$ represents the value of row i and column j of the output feature map after pooling, X represents the input feature map, and s represents the step size of the pooling operation (Stride), which is the distance of each sliding, where p is the size of the pooling kernel.

In the process of maximum pooling, the pooling kernel will slide on the feature map, take p pixels at a time, and output the maximum value as the pooled value [40]. Since the maximum pooling only takes the local maxima, it can effectively reduce the size of the feature map and preserve the main features of the image. At the same time, since the maximum pooling does not have any trainable parameters, it will not increase the complexity of the model.

We constructed a cross-entropy loss function. For a classification problem with N samples, let M be the number of categories, let $y_{i,j}$ be the true label of the i-th sample, j represents the label type, and $p_{i,j}$ is the probability that the i-th sample is predicted to be the j-th category. Then, the cross-entropy loss can be expressed as:

(18) $$L=-\frac{1}{N}\sum_{i=1}^{N}\sum_{j=1}^{M}y_{i,j}\log(p_{i,j})$$

During backpropagation, it is also necessary to calculate the gradient of each parameter to update the parameters. The optimization algorithm we used was stochastic gradient descent (SGD), and its update rule is:

(19) $$w=w-\alpha \frac{\partial L}{\partial w}$$

where w is the parameter and α is the learning rate.

The core algorithm of CNN is the convolution operation and the backpropagation algorithm. The convolution operation can be expressed in the form of discrete convolution. Assuming the input matrix X and the convolution kernel W, the output matrix Y of the convolution operation can be expressed as:

(20) $$Y_{i,j}=\sum_{p=1}^{m}\sum_{q=1}^{n}X_{i+p-1,j+q-1}W_{p,q}$$

where m and n are the sizes of the convolution kernels. Formula (20) represents the sliding window of the convolution kernel on the input matrix and performs weighted summation of the elements in the window [41].

The backpropagation algorithm was used to compute the gradient of the cost function with respect to each parameter. For each parameter, $w_{i.j}$, its gradient can be expressed as:

(21) $$\frac{\partial L}{\partial w_{i,j}}=\sum_{p=1}^{N_{l}}\sum_{q=1}^{M_{l}}\frac{\partial L}{\partial Y_{l,p,q}}\frac{\partial Y_{l,p,q}}{\partial w_{i,j}}$$

where $N_{l}$ and $M_{l}$ are the width and height of the output feature map of layer l, respectively. This formula represents the gradient of the cost function to each element in the output feature map, multiplied by the value of the corresponding element in the input matrix, and finally summed to obtain the parameter gradient.

When constructing the CNN model, in order to prevent overfitting, we used the dropout function, whose mathematical expression is:

(22) $$y_{i}=\left\{\begin{array}{cc} 0, & probability\;p\\ \frac{x_{i}}{1-p}, & \;\;\;\;probability\;1-p \end{array}\right.$$

During training, for an input x, the dropout function randomly sets the output of some of the neurons to 0. Let $x_{i}$ represent the output of the i-th neuron, where p is the dropout probability; that is, the probability of randomly setting the output of a neuron to 0, and p was set to 0.5 in this paper. At test time, dropout no longer randomly discarded the output of neurons, but kept the output of all neurons and divided the output by 1−p to keep the expected output unchanged.

Using the dropout function can prevent the model from overfitting and improve the generalization ability of the model [42]. It can be seen as a method of model averaging. By randomly discarding the output of neurons during training, the model randomly adopts different combinations of neurons, so that the model is more robust and less prone to overfitting.

### 5.2. Design of Federated Learning Algorithm for Insulator Fault Detection

We built the overall model of the federated experiment, where args. frac indicates the proportion or number of local models participating in training, args.num indicates the number of users participating in federated learning, E indicates the number of local training rounds, and η indicates the learning rate.

In the federated experiment, firstly, the dataset and user group were loaded. The dataset here was the dataset constructed in this paper, and the user groups were the groups into which the dataset was divided under independent identical distribution conditions. Then, the constructed models, CNN and MLP, were selected for insulator fault detection, and CNN and MLP were used as the global model. The global model is the overall total model, and the MLP and CNN we built can be considered at this point to be the pre-trained model as well as the overall model, which have weights at this point. We performed $\mathrm{copy}\;w_{0}$, and saved the weight of the global model into the global_weights variable, so that the weight parameters of the global model were passed to each local client. For each round, t, this experiment selected random user samples to update the local model: $w_{t+1}^{n}\leftarrow local$ wight $update\;(args.num,w_{t})$. This process is where the data passes through the neural network structure of the MLP or CNN we constructed and updates the weights of the local model. When the local model was updated, multiple local models clustered together, and the weights of the multiple local models were weighted and averaged. The weights of the global model: $w_{t+1}\leftarrow \frac{1}{n}\sum_{k=1}^{n}w_{t+1}^{n}$, were updated from Epoch 1 to E to obtain the final global model, and the model loss and accuracy were output. The detailed process is shown in Algorithm 1.


**Algorithm 1: Federated learning algorithm for insulator fault detection**

*Federated Learning (outline)*

**1. Input: **

dataset,usergroup


**2. Build Model:**
**3.**   *CNN or MLP***4.** *We build two global models CNN and MLP***5.**$\;\mathrm{Copy}\;w_{0}$ // *Get the current state of the model***6. Train:**   //*The following is the model training process***7.    For:** *each round t* =1,2, … **do****8.**    m←max⁡(args.frac⋅args.num,1) //*Get random sample of users*
**9. Local model update**
**10.**    $w_{t+1}^{n}\leftarrow localwightupdate(args.num,w_{t})$ // args.num = *n*
**11. Global model update**
**12.**    
$w_{t+1}\leftarrow \frac{1}{n}\sum_{k=1}^{n}w_{t+1}^{n}$
**13. For** *each local epoch*i *from 1 to E* **do****14.**    //*from 1 to E epoch updates the model gradient***15.**    w←w−η∇l      //*Generate the final global model***16. Output**   
*the model accuracy and loss of MLP or CNN*


## 6. Numerical Results

### 6.1. MLP Model

**Figure 5:**
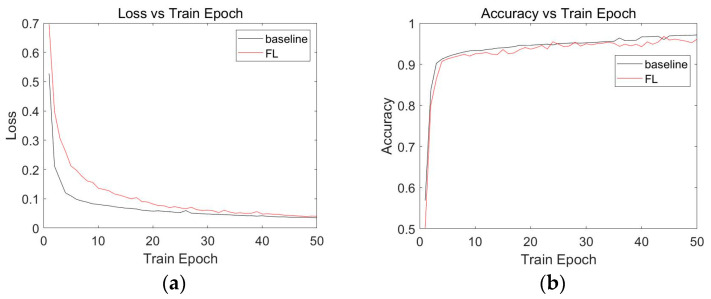
Experimental results of the MLP model. The baseline indicates a centralized detection algorithm and the FL indicates a federated learning-based detection algorithm. (**a**) MLP model loss. (**b**) MLP model accuracy.

The loss and accuracy profiles of the MLP model for the centralized insulator fault detection algorithm and the federated learning-based insulator fault detection algorithm are presented in Figure 5, where Figure 5a shows the loss profile of the MLP model, with the horizontal coordinate indicating the number of training epochs, and the vertical coordinate indicating the loss value. Figure 5b shows the accuracy curve for the MLP model, with the number of training epochs in the horizontal coordinate and the accuracy in the vertical coordinate. The black curves in Figure 5 uniformly represent the curves obtained by the model in the centralized insulator fault detection algorithm, and the red curves uniformly represent the curves obtained by the model in the federated learning-based insulator fault detection algorithm. From the experimental results in Figure 5a, it can be observed that the loss curves of the baseline and federated learning experiments in the MLP model showed an overall decreasing trend and gradually approached zero, with an increase in the Train Epoch. This indicates that the MLP model exhibited convergence in both the baseline and federated learning modes. As shown in Figure 5b, an increase in the Train Epoch led to a gradual improvement in the accuracy of both the baseline and federated learning experiments. When the Train Epoch approached 50 rounds, the model accuracy of the baseline was above 95%, and the model accuracy of federated learning was also over 95%, demonstrating acceptable model performance.

### 6.2. CNN Model

**Figure 6:**
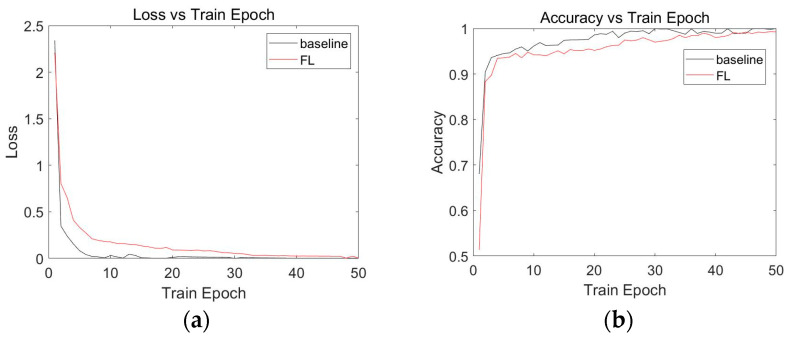
Experimental results of the CNN model. The baseline indicates a centralized detection algorithm and the FL indicates a federated learning-based detection algorithm. (**a**) CNN model loss. (**b**) CNN model accuracy.

The loss and accuracy profiles of the CNN model for the centralized insulator fault detection algorithm and the federated learning-based insulator fault detection algorithm are presented in Figure 6, where Figure 6a shows the loss profile of the CNN model, with the horizontal coordinate indicating the number of training epochs and the vertical coordinate indicating the loss value. Figure 6b shows the accuracy curve of the CNN model, where the horizontal coordinate indicates the number of training epochs, and the vertical coordinate indicates the accuracy. The black curves in Figure 6 uniformly represent the curves obtained by the model in the centralized insulator fault detection algorithm, and the red curves uniformly represent the curves obtained by the model in the federated learning-based insulator fault detection algorithm. The experimental results in Figure 6a demonstrate that both centralized baseline and distributed federated learning experiments for CNN also converge. According to the model accuracy in Figure 6b, it can be seen that in the CNN model, the baseline experiment accuracy reached a high level before the 10th Train Epoch and continued to approach 1 as the Train Epoch increased, while the accuracy of federated learning maintained a range of around 95%. When the epoch arrived at 50, the accuracy of federated learning approached 1, indicating that it has a high model accuracy performance.

We recorded the model accuracy of baseline and federated learning experiments for CNN and MLP models when the Train Epoch reached 50 rounds. As shown in Table 1, the accuracy of federated learning was not significantly different from that of the baseline experiment in both MLP and CNN models, and the model accuracy was maintained at a high level, demonstrating an acceptable insulator fault detection performance.

**Figure 7:**
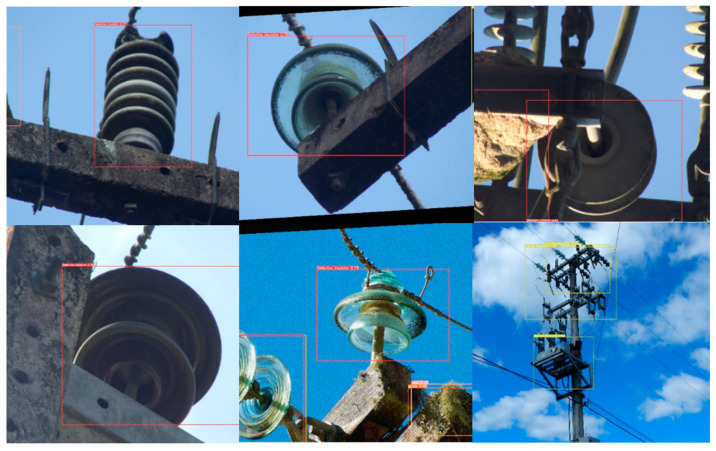
Insulator fault detection results.

Figure 7 illustrates the insulator fault detection performance based on the object detection algorithm.

### 6.3. Privacy Protection

**Figure 8:**
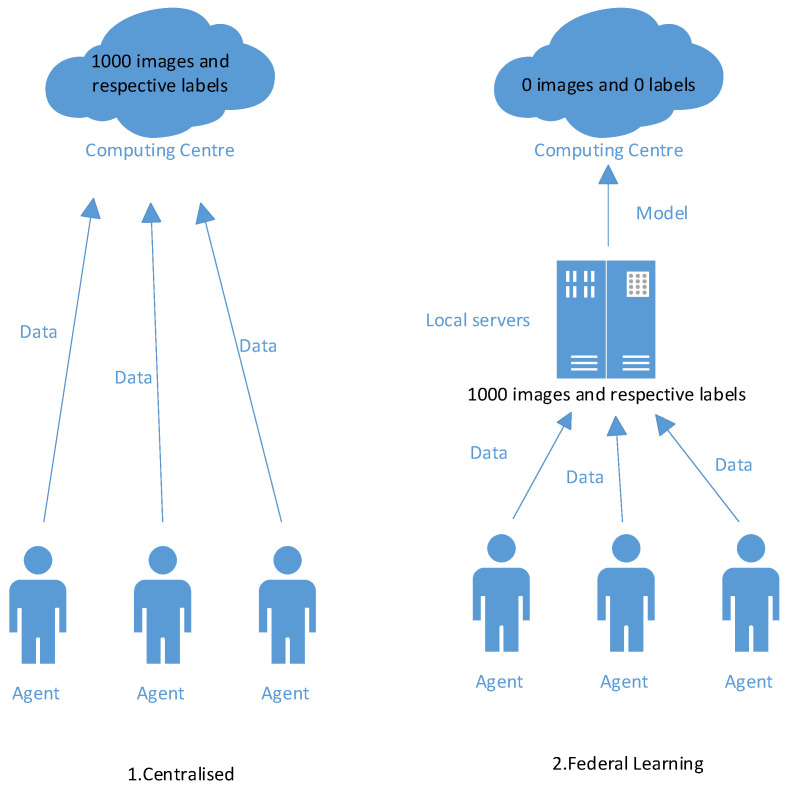
Privacy protection diagram. The left side is the centralized approach the right side is the federated learning approach. The 1000 images and corresponding tags contained in the dataset can be seen. In the centralized approach, this image and tag information is uploaded to the computing center, while in the federated learning approach, this information is only uploaded to the local server and not to the computing center.

Figure 8 presents a diagram of privacy protection. Under the centralized approach, all data were uploaded to the computing center, which receives an amount of data equal to the size of the dataset produced, a process that can cause privacy breaches. The federated learning detection approach differs from the centralized approach in that it is structured to have a local server to which all data are uploaded to generate the model. Only the generated models will be uploaded to the computing center. Whereas the centralized approach uploads all data to the computing center, the federated learning detection approach does not upload data to the computing center. The federated learning detection method has privacy protection.

## 7. Discussion

The federated learning model constructed in this article demonstrated excellent privacy protection capabilities, allowing for local model updates on user devices without sharing raw data, ensuring that sensitive user information remains distributed and secure. This approach effectively addresses privacy concerns and regulatory requirements, minimizing the risk of data breaches and unauthorized access to personal information. In addition, the use of model fusion technology further enhances the privacy guarantees of the federated learning model, making it suitable for privacy-sensitive areas.

Although federated learning is decentralized, the federated learning model established in this article has a competitive performance compared to centralized models. By participating in collaborative learning processes between client devices, the global model can leverage collective knowledge from different datasets, obtaining overall statistical characteristics. In addition, there is no need for data exchange between clients, which effectively protects personal data privacy. The experimental results showed that the federated learning model established in this article achieved an accuracy and generalization performance close to the centralized training methods, demonstrating its effectiveness in addressing challenges in distributed learning.

To improve the performance and robustness of the federated learning algorithm while maintaining the privacy of client data, a potential direction is to optimize neural network architecture and algorithm design specifically for the federated learning scenario. This may involve exploring techniques such as model compression, transfer learning, and adaptive optimization methods tailored for decentralized training. These methods aim to increase the efficiency and convergence speed of the federated learning process while minimizing communication overhead. Furthermore, considering the challenges posed by heterogeneous and non-IID (non-independent and identically distributed) data across client devices [43], future research efforts can focus on developing advanced federated learning algorithms that are more adaptive and robust to changes in data distribution and quality. Techniques such as personalized federated learning, federated transfer learning, and data augmentation can be explored to address the heterogeneity of client data and to improve the model’s generalization ability in real-world scenarios [44].

## 8. Conclusions

To address the privacy leakage issue caused by data uploading in insulator fault detection, we proposed a distributed insulator fault detection method based on federated learning. To validate the effectiveness of the distributed detection method, a dataset for insulator fault detection was constructed in this study, providing meaningful data for the community of distributed insulator fault detection. We constructed two model schemes, MLP and CNN, under the traditional centralized baseline and federated learning frameworks, and through experimental comparisons, the accuracy of the MLP model was 96.17% for the baseline experiment and 95.40% for federated learning, while under the CNN model, the accuracy was 98.91% for the baseline experiment and 97.67% for federated learning. Therefore, the distributed federated learning method can maintain an acceptable fault detection performance while ensuring user privacy. Additionally, federated learning requires a small amount of data and does not demand high local computational power, making it suitable for distributed scenarios in insulator fault detection. The next step in this research work is that we will further explore how much of a reduction in communication overhead this algorithm has. We will analyze the theoretical aspects and also try to improve the model of our classifier, after which we will attempt more advanced classification methods and more complex and advanced deep learning models, but still following the federal learning framework, as a way to improve the detection accuracy of the algorithm.

## Figures and Tables

**Table 1:** The model accuracy when the Train Epoch was set to 50.

	Experiment	Baseline	Federated Learning
Model			
MLP		96.17%	95.40%
CNN		98.91%	97.67%

## Data Availability

Not applicable.
